# Scaling the profile of life by function with SPIN

**DOI:** 10.1093/bioadv/vbag064

**Published:** 2026-02-19

**Authors:** Andrea Mancini, Vinh-Son Pho, Alessandro Bianchi, Gianluca Lombardi, Chujun Lyu, Alessandra Carbone

**Affiliations:** Department of Computational, Quantitative and Synthetic Biology (CQSB), IBPS, UMR7238, CNRS, Sorbonne Université, 75005 Paris, France; Department of Computational, Quantitative and Synthetic Biology (CQSB), IBPS, UMR7238, CNRS, Sorbonne Université, 75005 Paris, France; Department of Computational, Quantitative and Synthetic Biology (CQSB), IBPS, UMR7238, CNRS, Sorbonne Université, 75005 Paris, France; Department of Computational, Quantitative and Synthetic Biology (CQSB), IBPS, UMR7238, CNRS, Sorbonne Université, 75005 Paris, France; Department of Computational, Quantitative and Synthetic Biology (CQSB), IBPS, UMR7238, CNRS, Sorbonne Université, 75005 Paris, France; Department of Computational, Quantitative and Synthetic Biology (CQSB), IBPS, UMR7238, CNRS, Sorbonne Université, 75005 Paris, France; Institut Universitaire de France, Paris, 75005, France

## Abstract

**Motivations:**

Classifying hundreds of thousands of protein sequences by function remains a significant computational challenge. Building on the ProfileView method for identifying functional classes and subclasses, our goal is to achieve large-scale classification of proteins from extensive databases and ongoing high-throughput sequencing efforts, ultimately producing comprehensive sets of sequences that share the same function.

**Results:**

By applying deep learning techniques, SPIN learns discriminative patterns in functionally related sequences, allowing the classification of hundreds of thousands of sequences into a defined number of functional classes. SPIN offers an effective compromise between small, family-specific protein language models (pLMs) and computational cost, with a time complexity linear in the number of sequences. It enables the identification of family-specific conserved residues, providing insight into the functional nuances of protein subclasses. By enhancing the scalability of protein function predictors, SPIN advances our understanding of protein functions and their evolutionary relationships.

**Availability and Implementation:**

The data and code that support the findings of this study are publicly available at https://gitlab.lcqb.upmc.fr/andrea.mancini/SPIN.

## 1 Introduction

The comprehensive large-scale classification of available protein sequences represents a major challenge in bioinformatics. While many supervised machine learning (ML) tools have been developed based on known protein functions, the overall progress—both computationally and experimentally—in protein annotation remains limited to general functional categories. Understanding the specific activities of proteins requires distinguishing both the details of their actions and their interaction partners. This detailed level of annotation is often lacking, making experiments particularly challenging and costly, and underscoring the importance of unsupervised computational approaches.

Functional annotation of proteins relies on a variety of computational strategies, including sequence homology, profile similarity, structural modeling, feature-based methods, phylogenetics, and interaction data. Supervised ML models (Aggarwal and Hasija 2022) have advanced annotation efforts but remain constrained by limited and imbalanced training datasets. More recent approaches (Ryu *et al.* 2019, Zhou *et al.* 2022, Sanderson *et al.* 2023, Yu *et al.* 2023) improve performance yet still depend on predefined labels, typically drawn from incomplete systems like Gene Ontology (GO) (Dessimoz and Škunca 2017, The Gene Ontology Consortium 2021) or Enzyme Commission (EC) numbers (McDonald and Tipton 2023), which limit resolution—especially for multidomain proteins. Domain-based methods offer a complementary perspective by leveraging conserved structural and functional units (Orengo and Thornton 2005, Basu *et al.* 2009), as cataloged in resources such as InterPro (Blum *et al.* 2025), Pfam (Mistry *et al.* 2021), CATH (Dawson *et al.* 2017, Sillitoe *et al.* 2021), PANTHER (Mi *et al.* 2021), and MobiDB (Piovesan *et al.* 2023). To bypass the constraints of supervised learning, unsupervised methods like ProfileView (Vicedomini *et al.* 2022) cluster sequences into functional classes without prior labels, identifying key residues and subclass distinctions. By confidently defining accurate functional classes over a limited number of sequences, these methods provide a strong foundation for scaling annotations to vastly larger datasets. Building on this foundation, the objective here is to extend the power of classification tools toward large-scale functional categorization of large numbers of proteins—a need driven by the enormous and growing volume of sequence data from public databases and high-throughput sequencing efforts. The computational bottleneck in ProfileView, for instance, stems from a clustering procedure with $O(N^{2})$ time and memory complexity, where *N* denotes the number of sequences. As this step cannot be parallelized or batched, ProfileView is limited to ∼128 000 sequences on a system equipped with 64 GB of RAM. To overcome this limitation and enable truly large-scale analyses—spanning hundreds of thousands to millions of sequences within a protein family—we developed SPIN (Scaling the Profile of lIfe by fuNction). This deep learning architecture detects patterns among functionally related sequences first identified on smaller datasets, enabling the systematic assignment of large datasets of sequences into well-defined functional classes.

From enriched sets of sequences, SPIN will help to identify critical positions for performing functions by enriching the sets of sequences associated with subfamilies. Some of these conserved residues are shared across different subclasses, while others are unique to specific subclasses, providing essential insights into the functional nuances of these subfamilies. SPIN is expected to drive significant advancements in the large-scale functional annotation and classification of proteins, thereby deepening our understanding of protein functions and their evolutionary relationships. Notably, SPIN will be capable of identifying millions of sequences from databases like MGnify (Mitchell *et al.* 2020) and providing a primary classification of their protein domains.

SPIN is a DL architecture built on transfer learning. Instead of training from scratch—a process that is both time-consuming and resource-intensive—it capitalizes on both the classification of a core set of sequences and on the knowledge already acquired by ESM2 models during the pre-training phase. This is achieved by embedding protein sequences into the hidden representations provided by ESM2, which then serve as both the input and the backbone for the deep learning model.

## 2 Materials and methods

### 2.1 SPIN architecture

**Figure 1:**
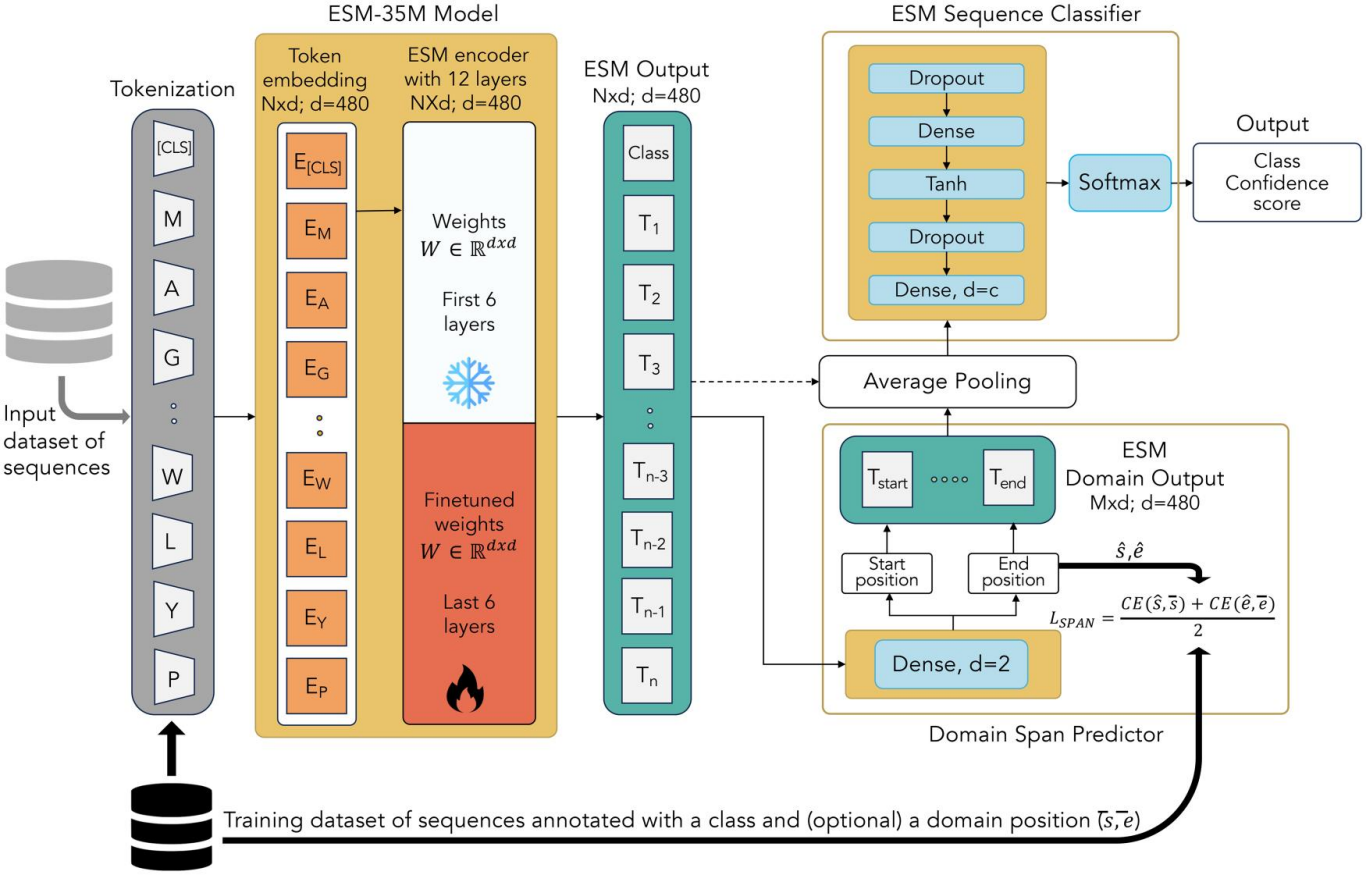
**SPIN architecture**. The input sequence, consisting of *n* residues, is transformed into an ordered list of tokens (grey) and processed by a small ESM2-35M model that has been fine-tuned on 6 (orange) out of 12 layers. Sequence tokens encoded with ESM2-35M (length N=n+1) are processed by removing the [CLS] token and feeding the remaining embeddings into a dense layer (cyan) to predict start and end domain positions (Domain Span Predictor module). The selected tokens lying within the identified domain boundaries and corresponding to a list of length M≤N, are averaged to form a 1×d vector, which is then processed by the classification head (ESM Sequence Classifier module). SPIN outputs a vector of scores (of dimension *c*, the number of classes) representing the confidence for the sequence to belong to a class. During training, the architecture takes a set of classified sequences together with an annotated domain (optional). If domain annotation is not given, then the Domain Span Predictor module is skipped and the entire ESM representation is averaged and passed to the classifier head.

As illustrated in Fig. 1, SPIN extends ESM2 and adapts the BERT question-answering framework (Devlin *et al.* 2018) to protein sequences. The architecture optionally predicts the span of a domain within a sequence and performs classification based solely on the identified region, enabling flexible use with or without domain annotations.

During training, SPIN takes as input a dataset of homologous sequences and, optionally, the annotated positions of a given domain. When domain boundaries are provided, this information is incorporated into the loss function to improve the model’s ability to localize domains. The training sequences are categorized into functional classes based on existing annotations and the desired level of functional specificity. The number of classes is defined by the user during model setup, enabling SPIN to flexibly adapt to any classification setting. This information is automatically saved in the configuration of fine-tuned model checkpoints.

During inference, SPIN processes sequences of arbitrary length without requiring prior knowledge of domain positions. When the model is trained to recognize a specific domain, it predicts the most likely start and end positions of the domain within each sequence and performs classification on the corresponding region. If multiple occurrences of the same domain are present within a sequence, SPIN is not expected to distinguish domain occurrences, and in this case, it will provide start and end positions with the best confidence scores and use the sequence lying within the two positions for classification. Note that SPIN checks that the start position precedes the end position, but it does not check for domain length. When the model is trained with no specified domain, inference is performed on the full-length sequences, i.e. on homologous proteins of comparable overall length, expected to share the same domain architecture—defined as multiple domains occurring in the same order.

Each amino acid sequence is tokenized and encoded by ESM2 to produce contextual embeddings, excluding the CLS token, which is not used in downstream processing. A Dense Domain Span Predictor outputs start ($T_{\mathrm{start}}$) and end ($T_{\mathrm{end}}$) positions of the span. It returns a logits vector (L×2) selecting positions with the highest scores. If $T_{\mathrm{end}}\leq T_{\mathrm{start}}$, the entire sequence is used. Embeddings within the predicted span are averaged across hidden dimensions to generate a one-dimensional representation, which is then passed to a classifier head for function prediction.

We used the 35M-parameter ESM2 model from HuggingFace (esm2_t12_35M_UR50D), which comprises 12 encoder layers with 480 hidden dimensions. To preserve low-level representations learned during pre-training and to reduce computational requirements during fine-tuning, the first six encoder layers were frozen, while the remaining six were fine-tuned for the downstream task. This configuration reflects the maximum number of trainable (non-frozen) layers supported by the available hardware (NVIDIA GPU A4000 with 16 GB VRAM). Because protein sequences vary substantially in length—and the attention mechanism’s memory requirements scale quadratically with sequence length—careful preprocessing was essential for efficient training. In our study, sequences longer than 1024 amino acids were excluded. This affected all three protein families analyzed: TRX: 28 601 → 28 151 sequences, CPF: 14 295 → 14 135 sequences, SH3: 11 148 → 8088 sequences. When domain annotations are absent, SPIN computes the average representation over all sequence tokens.

In the final stage of the architecture, the model applies average pooling over all token embeddings (either from the full sequence or the domain span, with the CLS token excluded as described above), followed by a classical classifier whose Softmax activation outputs normalized class probabilities that are used as class confidence scores. Our objective was to develop a method suitable for large-scale applications, capable of classifying hundreds of thousands to millions of sequences. Average pooling yields a compact, one-dimensional representation that supports efficient training and inference while avoiding the substantial computational overhead of residue-level modeling. Because ESM embeddings already capture contextual dependencies among residues, average pooling serves as an effective readout without discarding essential functional information. This design choice ensures both scalability and robustness in practice, whereas residue-level classifiers would be computationally prohibitive at such a scale and more susceptible to overfitting.

The architecture accepts input sequences via CSV, FASTA, or string lists, supporting both training and inference. When using a CSV file, each sequence entry should include a sequence ID and the amino acid sequence. In the training phase, it should also contain the start and end positions of the domain span (specified as integers) and a label for the class (e.g. ProfileView subtree ID). FASTA files or lists of sequences (provided as strings) are exclusively intended for inferring the protein functional class. During inference, if a CSV file is used, the output will include two additional columns: one for the predicted class and another for the corresponding probability score.

### 2.2 Hyperparameter values in SPIN

To determine the hyperparameters of SPIN, we used 14 135 sequences from the Cryptochrome/Photolyase Family (CPF), which contains the FAD domain (PF03441) and is classified in eight functional classes (see Figs S1–S3, available as supplementary data at *Bioinformatics Advances* online for characteristics of the CPF class distribution). We performed a grid search to identify a suboptimal yet well-balanced configuration, aiming to achieve strong performance while maintaining reasonable search efficiency and accounting for class imbalance. We separated model and training hyperparameters to reduce complexity. First, we tuned the model Dropout on 3 values: 0.0, 0.1, and 0.2, with a fixed training configuration. Then, we tested 16 combinations of learning rate ($10^{-4}$, $10^{-5}$), optimizer (Adam, AdamW), weight decay (0.0, 0.01), and scheduler (none, cosine annealing with warm-up). The best configuration used AdamW with a learning rate of $1\cdot 10^{-4}$, weight decay of 0.01, a cosine scheduler with warmup steps set to 10% of the total training steps, and a dropout rate of 0.1. The multi-loss function integrates two components: the cross-entropy loss for classification ($L_{\mathrm{class}}$) and the cross-entropy loss for span prediction ($L_{\mathrm{span}}$). A weighting of 70% is assigned to the classification term, reflecting the primary objective of achieving accurate class predictions. This choice prioritizes classification performance, as a more balanced weighting could potentially degrade it. Alternative weighting configurations were not further investigated. In $L_{\mathrm{class}}$, $\hat{y}$ and $\bar{y}$ denote the predicted and ground-truth class distributions, respectively, while ${\bar{w}}_{i,c}$ represents the class-specific weight (defined in Evaluation metrics) corresponding to the true class. The same notation applies to $L_{\mathrm{span}}$, where $\hat{s},\hat{e}$ are the predicted start and end positions from the Domain Span Predictor layer, and $\bar{s}$ and $\bar{e}$ are their ground-truth counterparts. The span loss is computed as the mean of the cross-entropy losses for the start and end position predictions. Here, CE(−,−) denotes the standard cross-entropy loss between a predicted probability distribution and its ground truth. The overall objective function is thus defined as:


$$L=0.7\cdot L_{\text{class}}+0.3\cdot L_{\text{span}}$$

$$\;L_{\text{class}}=-\sum_{i=1}^{N}{\bar{y}}_{i}\;\text{log}({\hat{y}}_{i}){\bar{w}}_{i,c},\;L_{\text{span}}=\frac{CE(\hat{s},\bar{s})+CE(\hat{e},\bar{e})}{2}$$

The CPF model was trained for 10 epochs using three random seeds, with batch sizes of 8 for training and 32 for validation and testing. Each training epoch required ∼13 minutes. Class weights (${\bar{w}}_{i,c}$) were applied to mitigate the effect of underrepresented classes. Model selection was based on the checkpoints with the highest validation ${F1}_{m-w}$ evaluated over 10 training epochs. As shown in Fig. S4, available as supplementary data at *Bioinformatics Advances* online, this training duration was sufficient to achieve near-optimal performance, while longer training resulted in overfitting and reduced generalization accuracy.

The same hyperparameter configuration—determined through grid search on the CPF family—was subsequently applied to the TRX and SH3 families.

### 2.3 Family-specific model tuning

For each protein family, an independent model was trained, validated, and tested on the same dataset using three different random seeds to account for the random fluctuations in the model’s layer parameters. Ideally, a training dataset for a given protein family should include a few thousand homologous sequences with known functional labels. These can be individual domains or full-length proteins with multi-domain architectures. Importantly, SPIN is designed to scale to large protein families and therefore does not require training on experimentally characterized sequences; instead, functional labels are typically obtained from computational annotation pipelines, including unsupervised approaches such as ProfileView.

During training, the known domain boundaries (start and end residue positions) of the sequences were incorporated into the loss function, enabling the model to be penalized when it incorrectly predicted the residues marking the beginning or end of domains. Performance was measured with weighted accuracy and macro-weighted F1-score to address class imbalance. Cross-entropy loss for DL models was adjusted via inverse frequency weighting. For the “Domain Span model,” a multi-loss function combined cross-entropy loss for class and span predictions, with a 70% weighting applied to the classification loss (as described above).

### 2.4 Bootstrap analysis of domain span predictions

When possible, identifying and isolating specific domains within sequences is recommended, as this enables the effective use of the domain span predictor within the SPIN architecture. To assess the performance of the domain span predictor in SPIN, bootstrap analysis was performed by repeatedly resampling the test set with replacement for 1000 times and computing domain boundary accuracy within a ±3-residue tolerance. Results were averaged over three random seeds to ensure robustness, and 95% confidence intervals (CIs) were estimated around the mean.

### 2.5 An experimental setup for comparative analysis

We compared SPIN architecture to a range of existing tools designed for classification tasks, spanning both traditional ML techniques and modern DL models. Traditional ML algorithms, valued for their interpretability and relatively low computational cost, were used as baselines. We tested Support Vector Machine (SVM), Gradient Boosting, Random Forest, and k-Nearest Neighbors (k-NN) to assess how well non-deep learning approaches capture class-specific patterns in protein sequences. For DL, we explored architectures tailored to the sequential and contextual nature of biological data, including Convolutional Neural Networks (CNNs), Long Short-Term Memory networks (LSTMs), Transformer-based models, and hybrid variants combining these techniques.

CNNs effectively detect local sequence patterns through convolutional filters. Our architecture, inspired by Kim (2014), uses a single convolutional layer with multiple kernel sizes (3, 5, 7), capturing patterns at various resolutions. Each feature map is processed with max-over-time pooling, and the pooled outputs are concatenated into a fixed-length vector and fed into a fully connected layer with dropout for classification.

LSTMs, and in particular Bidirectional LSTMs (BiLSTMs), capture long-range dependencies in sequences, essential for protein analysis where functional residues may be distant in the primary sequence but close in the folded structure. Our BiLSTM processes sequences in both forward and backward directions, concatenates hidden states, averages them across hidden dimensions, and passes the resulting embedding through two fully connected layers with dropout for classification.

For Transformer-based models, we used ESM2 (35M parameters, 12 encoder layers) as the backbone encoder (https://huggingface.co). A lightweight classification head (Dropout → Dense → Tanh activation → Dropout → Final Dense output layer) was added, with token representations mean-pooled to form a fixed-length embedding. We evaluated two settings: frozen, training only the classifier (backbone weights are kept fixed), and fine-tuned, updating the last six encoder layers during training.

We also explored hybrid models, combining ESM2-35M embeddings with CNN or BiLSTM classifiers to leverage contextual representations alongside local pattern detection (CNN) or sequential modeling (BiLSTM). Additionally, we incorporated a Domain Span strategy (detailed in Section 2.1), enhancing the fine-tuned ESM2-35M with explicit domain-aware modeling into the classification process.

For evaluation, hyperparameters were optimized via grid search, with distinct strategies for ML and DL models. ML models were tuned using five-fold cross validation. For DL models, due to the vast number of possible combinations and longer training times, we separated architectural choices from training hyperparameters. Optimizers were tailored per architecture—finding that Stochastic Gradient Descent (SGD) worked well for CNNs, while Adam/AdamW for ESM2 and LSTMs. During the grid-search phase, models were trained for five epochs; final evaluations were averaged from three random seed results obtained using 10 epochs. Two sequence encoding strategies were compared: one-hot encoding, using a fixed amino acid vocabulary (including an option for unknowns), and contextual embeddings from ESM2, where residues are dynamically represented based on sequence context. This latter approach yields much richer and more meaningful representations compared to the static one-hot encoding.

### 2.6 Evaluation metrics

To handle the multi-class and imbalanced nature of the datasets, we defined, for each class c∈C, true/false positives ($TP_{c}$, $FP_{c}$) and true/false negatives ($TN_{c}$, $FN_{c}$), with class weights $W_{c}=N/(|C|\cdot |s_{c}|)$, where $s_{c}$ is the support of class *c* and *N* is the number of training samples. We considered two weighted metrics, the Macro-Weighted F1-score:


(1) $${F1}_{m-w}=\frac{1}{\sum_{c=1}^{\left|C\right|}W_{c}}\sum_{c=1}^{\left|C\right|}W_{c}\cdot {F1}_{c}$$

where the F1-score for the class *c* is defined as $F1_{c}=2TP_{c}/(2TP_{c}+FP_{c}+FN_{c})$, and the Weighted Accuracy:


(2) $${\text{Acc}}_{w}=\frac{1}{\left|C\right|}\sum_{i=1}^{\left|C\right|}W_{c}\cdot \frac{TP_{c}+TN_{c}}{TP_{c}+TN_{c}+FP_{c}+FN_{c}}$$

### 2.7 Calibration errors

To evaluate the reliability of SPIN confidence estimates after training on a given protein family, we assessed probability calibration. Model outputs were calibrated using temperature scaling, as implemented in the Scikit library (Pedregosa *et al.* 2011), with parameters learned on the validation set. Calibration quality was quantified by computing the Expected Calibration Error (ECE) (Naeini *et al.* 2015) on the softmax probabilities and comparing values before and after calibration.

### 2.8 Discovery of conserved motifs

SPIN enables the extraction of conserved residues characteristic of protein subfamilies sharing a common function. By aligning domain sequences from each subtree, highly conserved, class-specific residues can be identified—likely contributing to functional differentiation. Motif discovery was performed using MEME [in MEME-suite v5.0.5; (Bailey *et al.* 2015)] with parameters: -nmotifs 5, -minsites 10, -minw 5, and -maxw 40. Motifs with an *E*-value < 1e-100 were retained. For overlapping motifs, the one with the lower *E*-value was selected. Motif alignment and position refinement were performed using TOMTOM (in MEME-suite) (Gupta *et al.* 2007), prioritizing alignments with the lowest *P*-values. While MEME provides a powerful framework for motif discovery, other tools—such as WebLogo (Crooks *et al.* 2004) or custom conservation analyses—can also be employed depending on the desired level of detail.

### 2.9 Datasets

The three sequence datasets analyzed in this study—representing the widely distributed protein families CPF, SH3, and TRX—were obtained from the UniProtKB (TrEMBL) database (UniProt Consortium 2025), with the fungal SH3 sequences additionally collected from NCBI (Goldfarb *et al.* 2025). Sequences include one of the expected Pfam domains (TRX: xref: PF00085, CPF: xref: PF03441, and SH3: xref: PF00018) and often are characterized by multi-domain architectures. Domain annotations for TRX and CPF were retrieved from UniProtKB and generated using MyCLADE (Ugarte *et al.* 2018, Vicedomini *et al.* 2021) for the two SH3 datasets.

Note that the sequences retrieved from UniProt were further filtered using both a clustering procedure to avoid high sequence similarity and ensure the feasibility of tree construction with ProfileView (mmseqs easy-cluster con—min-seq-id 0.5 -c 0.8). In fact, ProfileView further filtered the UniProt dataset by discarding sequences with low match scores to its models.

## 3 Results

SPIN is a deep learning architecture designed to differentiate sequences across a fixed set of classes and engineered to extend existing classification frameworks to much larger datasets. SPIN scales up the capability of current unsupervised and supervised approaches—which typically handle sets of thousands of sequences within a protein family—to hundreds of thousands or even millions. This scalability enables the enrichment of functionally similar sequences and facilitates the discovery of functional diversity by identifying residues responsible for specific activities. By leveraging the strengths of DL in extracting class-specific representations from large and complex datasets, SPIN provides an effective foundation for large-scale functional annotation.

### 3.1 The choice of a small PLM

SPIN is designed to efficiently process large numbers of sequences. Accordingly, employing a lightweight PLM is essential to ensure computational efficiency during both training and inference, without compromising predictive accuracy. To this end, we selected ESM2-35M and compared it with three widely used, substantially larger PLMs: ESM-2-650M (Lin *et al.* 2023), Ankh-base (Elnaggar *et al.* 2023) and ProtT5-XL (Elnaggar *et al.* 2021). The latter two models are based on T5 architecture. The models contain 650M, 450M, and 1.2B parameters, respectively, compared with the 35M parameters of ESM2-35M. This comparison was motivated by the need to assess whether ESM2-35M can match or surpass the performance of much larger models while offering dramatically improved computational efficiency. Notably, we found no other widely adopted PLM comparable in size to ESM2-35M. All PLMs were used as frozen backbones within the SPIN architecture and evaluated on the TRX dataset (with one seed), using the domain span predictor to assess their ability to extract functional signals. Training was performed with the same hyperparameters and number of epochs as those used for SPIN with ESM2-35M (Fig. S5, available as supplementary data at *Bioinformatics Advances* online). As shown in Table 1, ESM2-35M achieved highly competitive performance (validation and test $F1_{m-w}$ = 0.796) while being dramatically faster (0.53 s/iteration) than the larger models ProtT5-XL (4.43 s/iteration) and Ankh-base (3.90 s/iteration). Only the larger ESM2-650M model achieves higher performance, benefiting from an 18-fold increase in parameter number, which highlights the strong pre-training quality of the ESM2 family but comes at a substantial computational cost. Importantly, fine-tuning the three larger models was not feasible with our available hardware (single 16 GB VRAM GPU), whereas ESM2-35M comfortably fits within this constraint and further benefits from fine-tuning, achieving additional performance gains (Table 2). This combination of predictive accuracy, computational efficiency, and hardware feasibility makes ESM2-35M an ideal backbone for scaling functional annotation to very large protein datasets.

**Table 1:** Comparison of performance on TRX between different PLMs, serving as backbone in SPIN architecture. Best values per column are in bold.

PLMs classifier	PLM parameters	Validation		Test		Test span accuracy		Speed (s/iter) w/batch = 16	
		${\text{Acc}}_{w}$	${F1}_{m-w}$	${\text{Acc}}_{w}$	${F1}_{m-w}$	Start (±3)	End (±3)	Training	Test
ESM2-35M	35M (Frozen)	0.797	0.796	0.798	0.796	0.505	0.571	**0.53**	**0.52**
ESM2-650M	650M (Frozen)	**0.858**	**0.842**	**0.857**	**0.856**	**0.557**	**0.638**	4.52	4.51
ProtT5-XL-UR50-Half Encoder	1.2B (Frozen)	0.826	0.826	0.822	0.821	0.512	0.583	4.43	4.16
Ankh-base Encoder	450M (Frozen)	0.815	0.813	0.822	0.820	0.476	0.556	3.90	3.82

**Table 2:** Performance of SPIN on the TRX, SH3, and CPF protein datasets.^a^

	ProfileView		SPIN							
	Sequences	Classes	Training (80%)		Validation (10%)		Test (10%)		Test span accuracy (± 3)	
			${\text{Acc}}_{w}$	${F1}_{m-w}$	${\text{Acc}}_{w}$	${F1}_{m-w}$	${\text{Acc}}_{w}$	${F1}_{m-w}$	Start	End
TRX	28 151	8	0.985 ± 0.033	0.985 ± 0.033	0.913 ± 0.006	0.913 ± 0.006	0.906 ± 0.003	0.906 ± 0.003	0.823 ± 0.002	0.819 ± 0.002
SH3	8 088	7	0.999 ± 0.0003	0.999 ± 0.0003	0.902 ± 0.0003	0.902± 0.0003	0.887 ± 0.001	0.887 ± 0.001	0.969 ± 0.002	0.980 ± 0.002
CPF	14 135	8	0.988 ± 0.0002	0.987 ± 0.0002	0.982 ± 0.0002	0.982 ± 0.0002	0.977 ± 0.003	0.976 ± 0.003	0.963 ± 0.002	0.909 ± 0.005

a Reported values represent the mean ± standard deviation across three independent training runs initialized with three random seeds. Ground truth labels were defined based on the ProfileView classification.

### 3.2 Performance across representative protein families

To evaluate the generality and robustness of SPIN, we analyzed its performance across three representative protein families—CPF, TRX, and SH3—which together encompass diverse structural folds and functional contexts. The CPF family comprises cryptochromes and photolyases, performing diverse light-dependent functions ranging from DNA repair to photoreception and transcriptional regulation, providing a challenging test case for functional differentiation within homologous sequence space. The TRX family represents well-characterized oxidoreductases with conserved catalytic motifs. SH3 domains mediate protein–protein interactions through distinct binding surfaces. This selection therefore provides a broad testbed, spanning different sequence lengths, domain architectures, and evolutionary conservation patterns, allowing us to assess the model’s ability to capture function-specific features under varying levels of sequence diversity.

Table 2 reports the training, validation, and test performances of SPIN across the three protein families. SPIN consistently achieves high ${\text{Acc}}_{w}$, ${F1}_{m-w}$, and domain span accuracy, confirming its ability to capture class-specific features. Performance remains stable between the training and validation sets, indicating effective convergence and minimal overfitting within the first few epochs.

Since SPIN was optimized on the CPF family, we present results for the TRX and SH3 families to illustrate the model’s performance and the nature of the information it captures. A detailed analysis of SPIN for the three proteins is reported in Figs S1–S3 and S6–S8, available as supplementary data at *Bioinformatics Advances* online.

### 3.3 SPIN on thioredoxin, a widespread protein family

Starting from a ProfileView-based classification of thioredoxins (TRX), a protein family widely distributed across all domains of life, SPIN expands the initial set of labeled sequences by incorporating large numbers of additional sequences, enriching existing functional classes. Our results show that evolution uniquely shapes distinct functional signals within TRX-specific subclasses.

**Figure 2:**
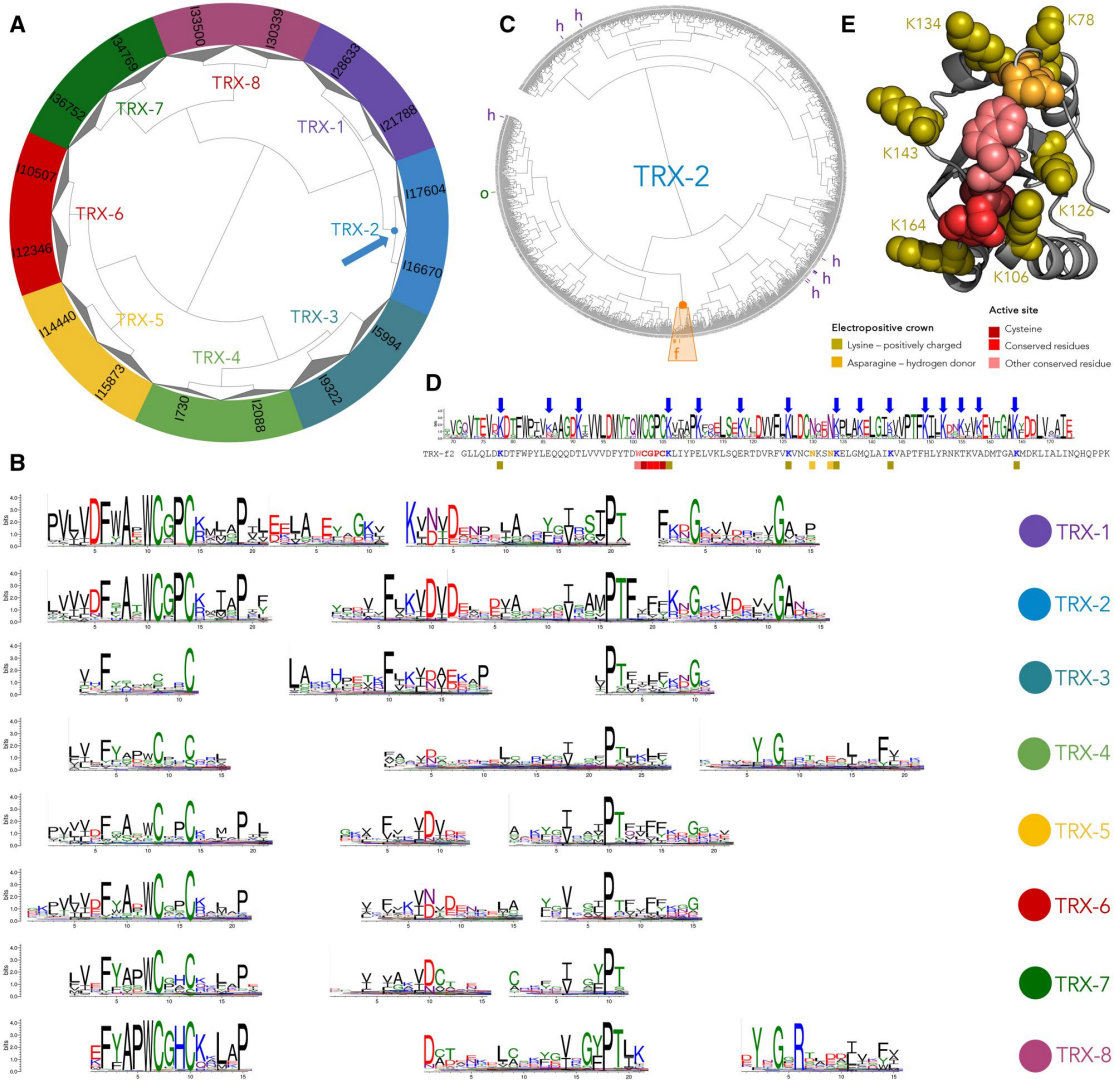
**Analysis of the TRX family and TRX type f subfamily.** (A) Functional tree of the TRX family generated with ProfileView, identifying eight subclasses at depth 3. The tree is visualized using iTOL (Letunic and Bork 2021). (B) Conserved motifs are computed with MEME from the sequence alignments of the eight subtrees in panel (A). Motifs are aligned with TomTom. (C) Subtree constructed from sequences in subtree TRX-2 from panel (A) (blue root). It contains known TRX type f sequences from *Chlamidomonas reinhardtii* (e.g. TRX-f2; orange root). Other TRX sequences of types h and o are also indicated. Visualization done with iTOL (Letunic and Bork 2021). (D) Conserved profile generated using WebLogo (https://weblogo.berkeley.edu/logo.cgi) from the TRX-2 subtree (in orange, panel C) containing type f sequences. The *C. reinhardtii* TRX-f2 sequence in the tree is aligned with the profile. Colored squares mark conserved residues described in panel (E), and blue arrows indicate conserved lysines in the profile. (E) TRX-f2 structure (PDB 6i1c), showing functional residues for the *C. reinhardtii* sequence: the extended disulfide bond motif (WCGPC, red tones), a positively charged crown surrounding the active site (eight lysines, olive green), and two asparagines (orange). All other residues are grey.

To quantitatively assess the effectiveness of this strategy, we evaluated SPIN on a ground truth comprising 28 151 TRX sequences (Table 2), labeled into eight functional classes using ProfileView. These classes were identified by embedding the sequences into the ProfileView multidimensional space, where functional similarity is reflected by spatial proximity. As illustrated in Fig. 2A, hierarchical clustering of this space reveals eight subclasses corresponding to subtrees at depth 3.

#### 3.3.1 Large classes of TRX sequences are important for training

SPIN was trained on 80% of the labeled TRX dataset (22 520 sequences) and evaluated on the remaining 20% (5631 sequences) on whether or not it preserved class distribution (Figs S2 and S3, available as supplementary data at *Bioinformatics Advances* online). SPIN achieved a weighted accuracy of 0.9169 and a macro-weighted F1-score of 0.9140 on the validation set and 0.915 on the test set using the best random seed. As expected, the class size used during training (Fig. S3, available as supplementary data at *Bioinformatics Advances* online) influences the model’s performance, with smaller classes yielding lower recall scores. Despite applying inverse frequency weights to the cross-entropy loss, DL remains biased toward larger classes, which dominate the training statistics (Fig. S6, available as supplementary data at *Bioinformatics Advances* online).

Table 3 shows that SPIN performs better with increasing training set size, with all results computed on the same fixed validation and test sets. The validation weighted accuracy and macro-weighted F1-score plateau at 0.90, and test performance at 0.89, when training on 60% of the 28 151 TRX sequences—similar to results obtained with 80% of the data (Fig. 2). Even with only 10% of the dataset, the model reaches weighted accuracy and macro-weighted F1-scores above 0.80.

**Table 3:** SPIN performance with varying training set sizes.^a^

Training set size	Validation		Testing	
	${\text{Acc}}_{w}$	${F1}_{m-w}$	${\text{Acc}}_{w}$	${F1}_{m-w}$
282 (1%)	0.6949	0.6827	0.6997	0.6859
2 815 (10%)	0.8508	0.8494	0.8291	0.8275
5 630 (20%)	0.8599	0.8584	0.8624	0.8621
11 260 (40%)	0.8943	0.8937	0.8874	0.8868
16 890 (60%)	0.9074	0.9076	0.8925	0.8918
22 520 (80%)	0.9125	0.9125	0.9056	0.9058

a Results, based on 28 151 TRX sequences, report weighted accuracy and macro-weighted F1-scores computed on fixed validation and test sets. Training was conducted on progressively larger subsets of the dataset (percentage of the full dataset shown in parentheses).

For domain span prediction, we achieved a weighted accuracy score of 0.82 when allowing a stringent ±3-position tolerance at domain borders (Table 2). Without tolerance, weighted accuracy dropped to 0.47 for start positions and 0.60 for end positions (Fig. S7, available as supplementary data at *Bioinformatics Advances* online). Bootstrap distributions and confidence intervals (CI) confirm low variance and stable predictions with a mean of 0.82 and CI = [0.81–0.83] for start positions and a mean of 0.83 in [0.82–0.84] for end positions (Fig. S8, available as supplementary data at *Bioinformatics Advances* online).

#### 3.3.2 ProfileView versus SPIN ESM2-35M embeddings

**Figure 3:**
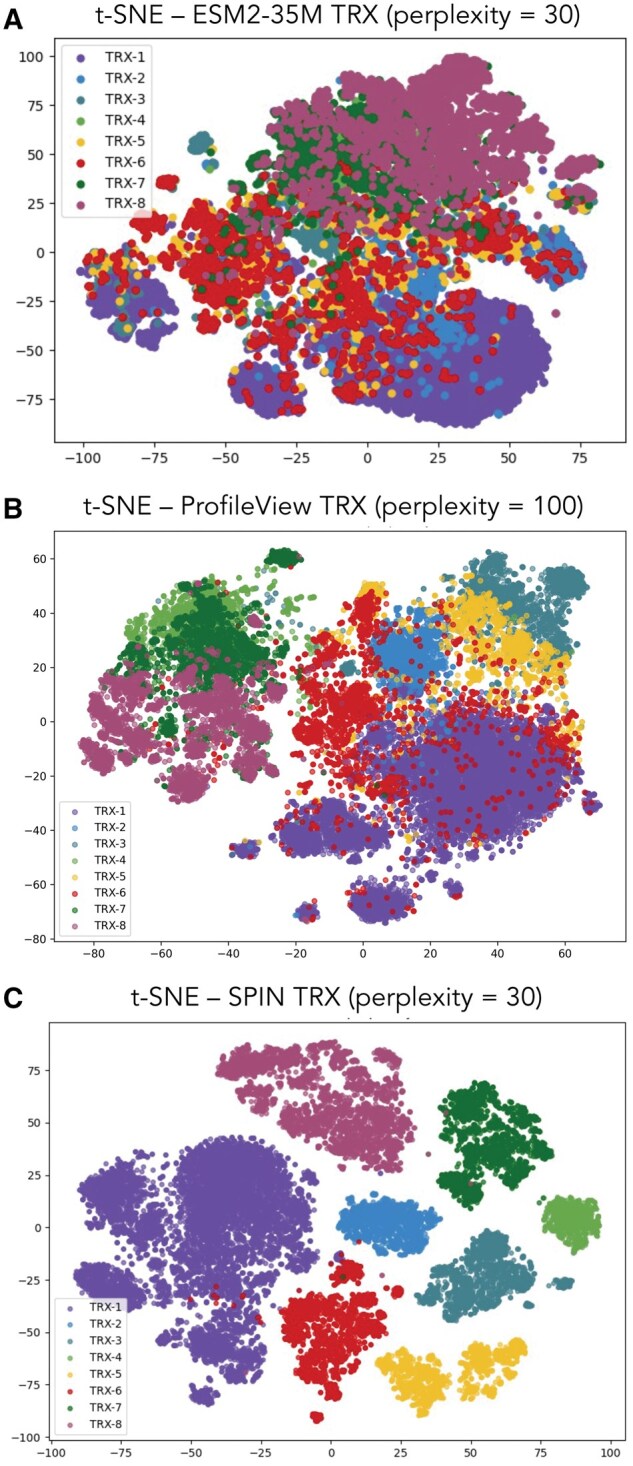
**Visual representation of the 22 520 TRX sequences used in training SPIN.** Sequences are shown in (A) the ESM2-35M embedding space, generated with perplexity at 30, 1000 iterations and PCA; (B) the ProfileView embedding space, generated with the t-SNE algorithm, with perplexity at 100, 1000 iterations and PCA; and (C) the SPIN ESM2-35M embedding space after fine-tuning, generated with perplexity at 30, 1000 iterations and no PCA.

We compared ProfileView embeddings of the 22 520 training sequences (Fig. 3B) with their corresponding ESM2-35M embeddings obtained in SPIN (Fig. 3C). ProfileView embeddings reside in a multidimensional space of a few hundred dimensions (∼200) specifically designed to extract functional information, whereas SPIN embeddings were generated using 480-dimensional vectors from the ESM2-35M model after hyperparameters tuning and fine-tuning. The SPIN vector representing a sequence is obtained by applying average pooling to the ESM Domain output (Fig. 1), a necessary step for entering the classification head. Note that ProfileView encodes sequences by focusing on the TRX domain within; similarly, SPIN considers its predicted domain span (Fig. 1).

SPIN clearly learns to sharply separate sequences in its 480-dimensional space. In contrast, the visualization of the pure ESM2-35M embeddings without fine-tuning (Fig. 3A) highlights the substantial improvement gained through fine-tuning, beyond what is already present in the pre-trained sequence representations.

#### 3.3.3 Functional signals across TRX subclasses

An analysis of the eight TRX functional classes revealed class-specific conservation profiles (see Fig. 2B). Within classes TRX-1 and TRX-2, motif alignment highlights two globally conserved cysteines and a proline (Fig. 2B). These classes include sequences from the microalga *Chlamidomonas reinhardtii* and other plants, where the CGPC disulfide bond motif is known to play a critical functional role. This motif, located at positions 31–34 in the *C. reinhardtii* TRX-f2 sequence, corresponds to residues highlighted in dark red in the associated PDB structure (Fig. 2E and F).

#### 3.3.4 Functional signals within TRX subclasses: the identification of specialized functions

SPIN enrichment in the number of sequences that share highly similar functions is critical for uncovering functional signals that are only detectable within very narrow functional niches, where subtle but biologically meaningful sequence patterns emerge only when sufficient numbers of closely related sequences are available.

This principle is well illustrated by the TRX type-f protein from *C. reinhardtii*. TRX-f2 belongs to the TRX-2 subtree, which comprises 1699 sequences (Fig. 2A). Previous structural studies identified an extended active-site disulfide motif, WCGPCK, and showed that residues flanking the catalytic cysteines contribute to an extended three-dimensional motif controlling thioredoxin reactivity (Mavridou *et al.* 2014, Lemaire *et al.* 2018). Using SPIN in combination with MEME, we recovered this extended motif as highly significant for the TRX-2 subtree (Fig. 2B), confirming that large-scale functional grouping preserves fine-grained catalytic features.

Importantly, by further restricting the analysis to deeper branches of the tree and focusing on sequences closest to *C. reinhardtii* TRX-f2 (the orange-rooted subtree in Fig. 2C), we were able to construct a TRX-f2–specific profile (Fig. 2D). While the MEME motifs identified at the level of the broader functional class TRX-2 (Fig. 2B) do not show evidence of conserved lysine residues, this finer-grained subclustering revealed an unexpectedly large number of conserved lysines, suggesting a role in substrate recruitment. The absence of lysine conservation at the TRX-2 class level, contrasted with their enrichment in the TRX-f2–specific profile, indicates that these residues are not generic features of the TRX-2 function but rather hallmarks of a more specialized functional niche. Notably, these lysines do not correspond to a single strictly conserved position; instead, they appear at multiple positions across the sequences within the subtree, forming a distributed yet functionally coherent pattern that would be difficult to detect without sufficient sequence depth.

Intersecting the conserved lysines identified in the TRX-f2 profile with those present in the *C. reinhardtii* sequence highlighted six lysines, along with two asparagines, previously reported as unique to TRX-f2 and responsible for forming a positively charged crown around the enzyme’s active site (Lemaire *et al.* 2018) (Fig. 2E). In Fig. 2E, motif residues are shown in red tones and lysines in olive green on the TRX structure. Together, this analysis demonstrates how scaling functional classification with SPIN enables the enrichment of closely related functional sequences, which in turn allows the identification of subtle, distributed sequence features underlying specialization within very narrow functional subclasses.

### 3.4 Analysis of the SH3 protein family and representational augmentation across clades

The SRC Homology 3 (SH3) domain is a small (∼60 amino acid) module commonly found in signaling proteins, where it mediates interactions with adaptor proteins and tyrosine kinases. SH3 domains are widespread across proteomes; e.g. the human genome encodes around 300 such domains.

**Figure 4:**
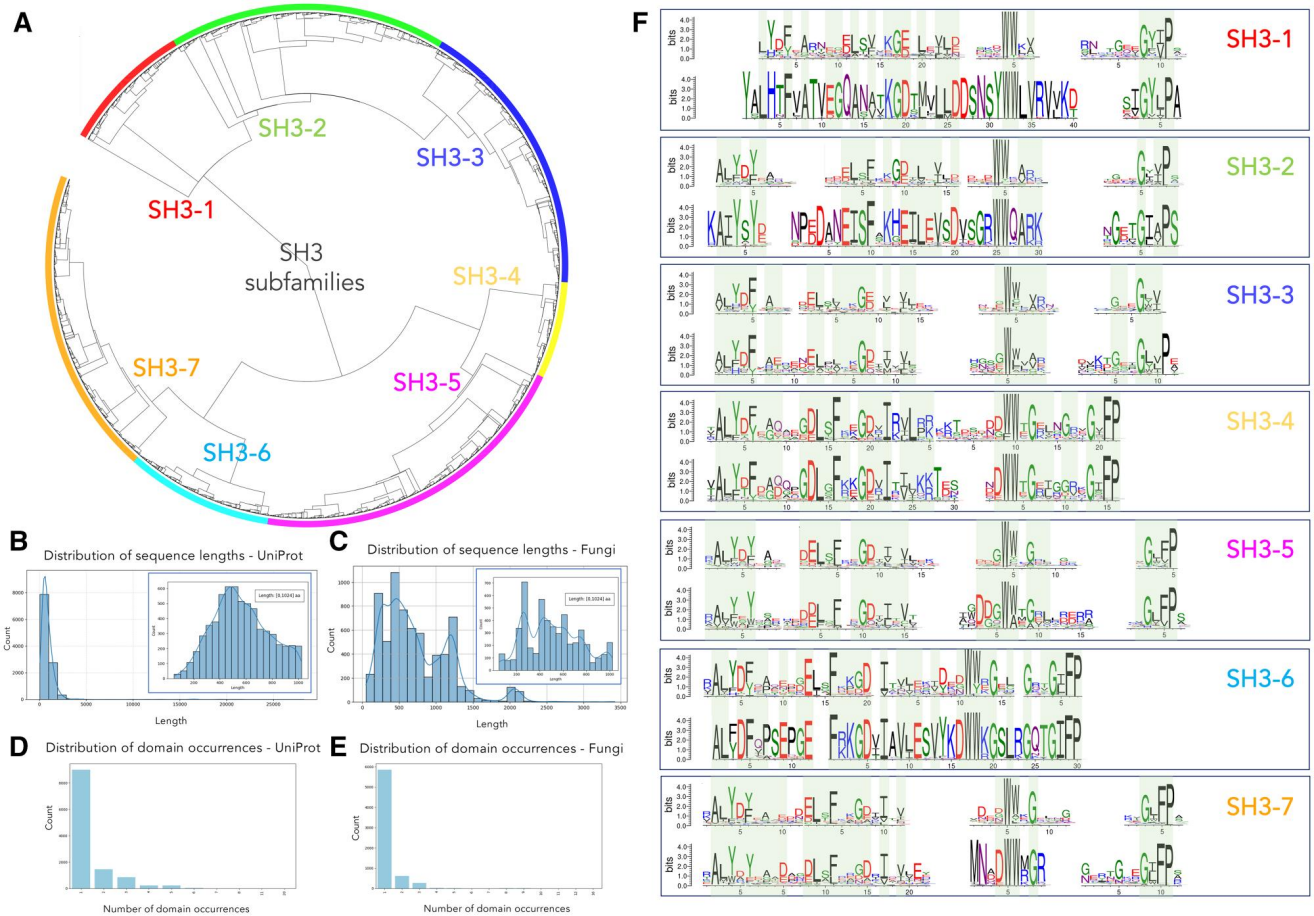
**Analysis of the SH3 protein family.** (A) A set of 11 148 UniProt sequences containing SH3 domains was analyzed with ProfileView to define a classification of the sequences in seven distinct functional classes. They correspond to the colored subtrees of ProfileView functional tree. The dataset contains 8088 sequences of length <1024 amino acids which have been used in training SPIN. (B) Distribution of sequence lengths for the set of SH3 containing UniProt sequences. In the inset, distribution of the UniProt sequences of length <1024 amino acids used to train SPIN on SH3 domain classification. (C) Distribution of sequence lengths for the set of SH3 containing NCBI Fungi sequences. In the inset, distribution of the NCBI sequences of length <1024 amino acids used to test SPIN on SH3 domains. (D) Distribution of SH3 domain occurrence in the UniProt dataset. (E) Distribution of SH3 domain occurrence in the Fungi dataset. (F) Comparison of MEME conservation motifs across the seven functional classes of SH3. The reference classification of the SH3 domains is illustrated in (A). For each subtree of UniProt sequences in (A), the MEME motif for these training sequences (top) is compared to the MEME motif derived from the Fungi sequences classified to belong to the same functional class during testing (bottom). A green background highlights conserved positions in each aligned pair of motifs, with conservation concerning physico-chemical amino acids properties.

We evaluated SPIN on the task of classifying SH3 domains embedded in proteins with complex architectures and originating from diverse species. As ground truth, we used 8088 UniProt sequences, of at most 1024 amino acids in length, each containing one or more SH3 domains, classified with ProfileView in seven classes. The functional tree generated with ProfileView (Vicedomini *et al.* 2022) is shown in Fig. 4A.

During inference, SPIN scans all potential SH3 occurrences within a sequence and selects the one with the highest probability of being a true SH3 domain, assigning the sequence to a functional class accordingly. Figure 4B and D show the distribution of sequence lengths and SH3 domain counts in the training dataset (see Fig. S1, available as supplementary data at *Bioinformatics Advances* online for the testing dataset). Table 2 (Fig. S7, available as supplementary data at *Bioinformatics Advances* online) summarizes the model’s performance, demonstrating high domain-span accuracy despite the complexity of classifying small domains within long protein sequences. Bootstrap distributions and CI confirm low variance and stable predictions with a mean of 0.97 and CI = [0.96–0.98] for start positions and a mean of 0.98 in [0.97–0.99] for end positions (Fig. S8, available as supplementary data at *Bioinformatics Advances* online). The SH3 family exhibits accuracies roughly 10% higher than TRX, likely reflecting lower variance in domain lengths (Fig. S1, available as supplementary data at *Bioinformatics Advances* online), which facilitates model learning. Dataset size and sequence diversity (Fig. S2, available as supplementary data at *Bioinformatics Advances* online) may also contribute to these trends.

The partition into functional classes provides the learning framework for SPIN, enabling it to identify conserved, class-specific sequence patterns that remain informative even under substantial evolutionary divergence, thereby augmenting the explored sequence space in novel evolutionary clades.

To assess its broader applicability, we applied SPIN to a separate dataset of 6910 fungal sequences containing SH3 domains, none of which were used for training. The fungal dataset exhibited a similar distribution of sequence lengths and SH3 occurrences as the UniProt set (compare Fig. 4B and D and Fig. 4A and C). We tasked SPIN with assigning each sequence to one of the seven predefined classes. Despite the evolutionary distance between fungi and the species represented in UniProt, SPIN was able to project these new sequences into the learned representation space, effectively extending the training-derived representation to a distinct evolutionary clade.

For each class, MEME motifs derived from UniProt sequences were compared with motifs from fungal sequences (Fig. 4F), showing strong motif conservation across datasets. This conservation indicates that SPIN extracts class-specific sequence features that persist across evolutionary distances.

As an additional test, we independently classified the Fungi dataset using ProfileView. Three-dimensional t-SNE projections of the ProfileView functional spaces for UniProt (Fig. S9A, available as supplementary data at *Bioinformatics Advances* online) and Fungal (Fig. S9B, available as supplementary data at *Bioinformatics Advances* online) sequences show clear class separation. SH3-1, -2, and -3 cluster together in one region, while SH3-4, -5, -6, and -7 occupy another, mirroring the division seen in Fig. 4A. The preservation of this separation in the fungal sequences further supports the weighted accuracy of SPIN’s classification.

Interestingly, SPIN assigned most fungal sequences to six of the seven classes and classified very few sequences as SH3-1. Although this class is strongly underrepresented in the fungal dataset, SPIN still detects the conserved sequence patterns defining SH3-1. MEME motifs derived from the SH3-1 fungal sequences closely match the corresponding UniProt motifs, indicating that the conserved features defining this class are preserved and remain sufficient for reliable classification even with few evolutionarily distant examples.

### 3.5 Reliability of SPIN confidence scores

To evaluate the reliability of SPIN confidence estimates after training on a given protein family, we assessed probability calibration by measuring the Expected Calibration Error (ECE) (Naeini *et al.* 2015) on softmax probabilities before and after calibration. All models exhibited low initial ECE values on the validation set, with differences emerging primarily at the second decimal place. Temperature scaling consistently reduced calibration error across families. Specifically, for the TRX model, ECE decreased from 0.0403 to 0.0161 on the validation set and from 0.0359 to 0.0083 on the test set. The CPF model showed a reduction from 0.0067 to 0.0039 on validation and from 0.0099 to 0.0085 on the test set. Similarly, the SH3 model improved from 0.0643 to 0.0199 on validation and from 0.0819 to 0.0400 on the test set.

To further assess the behavior of calibrated confidence scores under distributional shift, we evaluated each model on sequences drawn from protein families different from the one used for training. For this analysis, we sampled 1000 sequences from each non-matching family and examined the resulting calibrated probability distributions (Fig. S10, available as supplementary data at *Bioinformatics Advances* online). As expected, predictions for non-matching families exhibited left-skewed probability distributions, in contrast to the right-skewed distributions observed for test sequences belonging to the corresponding trained family. Notably, for each model, a confidence threshold emerged near the intersection of the two distributions, providing a natural separation between in-family and out-of-family predictions. This threshold was ∼0.8 for both the TRX and SH3 models and higher for the CPF model, at around 0.9.

Finally, after calibration, we performed an additional experiment on the TRX family to assess the abstention capability of SPIN when confronted with pseudo-random sequences. To generate this dataset, each TRX test sequence was first segmented into contiguous chunks of five amino acids, preserving overall amino acid composition while disrupting domain-level structure; the chunks were then randomly shuffled within each sequence. As shown by the confusion matrix (Fig. S11A, available as supplementary data at *Bioinformatics Advances* online), SPIN exhibits a prediction bias toward TRX-4 when evaluated on these out-of-distribution sequences. Notably, TRX-4 is the least represented TRX class (Fig. S3, available as supplementary data at *Bioinformatics Advances* online) and effectively functions as a garbage class, absorbing the majority of reshuffled sequences (Fig. S11B, available as supplementary data at *Bioinformatics Advances* online). The corresponding distribution of calibrated probability scores (Fig. S11C, available as supplementary data at *Bioinformatics Advances* online) displays high entropy, consistent with the random nature of the input data. In contrast, the calibrated score distribution for the original TRX sequences (Fig. S11D, available as supplementary data at *Bioinformatics Advances* online) shows a clear separation from that of the reshuffled data, indicating strong discriminative capacity. Within this setting, applying a confidence threshold, as discussed above, enables effective separation of confident predictions from uncertain ones. For example, using a threshold of 0.8 correctly identifies 81% of the reshuffled sequences as unknown. Consistent with this behavior, performance on the reshuffled dataset is low, with an accuracy of 0.0443, an F1 score of 0.0269, a span start accuracy of 0.0458, and a span end accuracy of 0.0301. A similar analysis was conducted by fully reshuffling amino acids within each TRX test sequence (Fig. S11E–H, available as supplementary data at *Bioinformatics Advances* online). Performance in this dataset is low, with an accuracy of 0.0440, an F1 score of 0.0281, a span start accuracy of 0.0447, and a span end accuracy of 0.0241. Interestingly, SPIN assigns fewer high-confidence predictions to sequences generated via 5-amino acid chunk reshuffling than to fully reshuffled sequences, with 19% versus 25% of sequences receiving calibrated scores above 0.8, respectively. This suggests that SPIN more effectively rejects pseudo-random sequences that retain a minimal protein-like local context: short coherent motifs may be detected, but their disrupted global arrangement yields conflicting evidence across the sequence, which is reflected in lower calibrated confidence and improved abstention compared to fully randomized inputs.

### 3.6 Comparison with other computational approaches

In Table S1, available as supplementary data at *Bioinformatics Advances* online, we compared SPIN with both classical ML methods and supervised DL architectures dedicated to multiclass classification. Although DL has become the dominant paradigm, including ML baselines remains essential: these models have historically been the standard in protein function prediction, they are computationally efficient, and their explicit reliance on predefined features provides a useful contrast with representation learning approaches. Testing them on functionally classified sequences allowed us to assess whether simpler, widely accessible models could still capture discriminative signals. As expected, the limited ability of ML algorithms (SVM, Gradient Boosting, Random Forest, K-NN) to autonomously extract relevant features constrained their statistical performance. In contrast, DL architectures (CNN, bidirectional LSTM, and Transformers) offered stronger representational power but were prone to overfitting and biased toward classes with greater training support. Overall, ESM2-35M fine-tuned and combined with the Domain Span module achieves the best performance across all validation and test metrics, outperforming all competing ML and deep learning baselines, while the comparatively weaker performance of its frozen counterpart demonstrates the necessity of fine-tuning to reach optimal performance.

## 4 Computational complexity analysis

The complexity of SPIN’s transformer architecture, driven by the parallelism of the multi-head attention mechanism, scales linearly with the number of sequences and quadratically with sequence length due to the self-attention computation (Huang *et al.* 2023). To prevent GPU memory overflow, ESM2 was tested only with sequences up to 1024 tokens (Rives *et al.* 2021). Consequently, for SPIN, the overall computational complexity is $O(N\cdot L^{2})$ and since L=1024 is effectively constant, runtime scales linearly with *N*, the number of sequences. During testing, inference times were recorded at 33 ms for a single sequence and 1.05 s for a batch of 32 sequences. Training was conducted on an NVIDIA GPU A4000 with 16 GB VRAM, using 10 epochs per training run, with an average training time of ∼25 minutes per epoch on the TRX dataset. These results demonstrate that SPIN achieves supervised classification using a compact protein language model while maintaining a reasonable computational cost for both training and inference.

To assess large-scale performance, we conducted a classification experiment on 202 644 sequences from the TRX family. Inference required ∼110 minutes with a batch size of 32, confirming the linear scaling of SPIN with respect to the number of sequences. Unlike ProfileView, SPIN fully exploits GPU-accelerated matrix operations and algebraic computations, substantially enhancing scalability and efficiency.

## 5 Discussion and conclusions

Having a robust classification method such as SPIN is essential for enhancing our ability to detect and group sequences sharing common functions—a task of increasing importance as we face the challenge of classifying millions of proteins. Building on methodologies like ProfileView, which efficiently identifies functional classes and subclasses from thousands of sequences, SPIN leverages these insights to enrich the representation of sequences with similar roles for arbitrarily large sets.

However, this approach currently circumvents the main computational challenge of directly handling millions of sequences in a dynamic classification process. The number of subclasses is fixed upfront, derived from ProfileView’s analysis of a smaller representative dataset. Ideally, a classification system should ingest millions of sequences directly and autonomously discover subclasses by capturing subtle differences in their encoded functional signals.

ProfileView nevertheless remains critical in providing a sufficiently large and balanced training set. As shown in our analysis, DL models tend to specialize toward the most frequently observed classes, making adequate representation of all functional categories essential for achieving high accuracy in SPIN.

Once the number of functional classes is defined and a coherent training dataset is available, SPIN can scale without intrinsic limits on the number of sequences to classify; the only practical constraint is computational time. Based on our measured inference speed, SPIN can process around 1 million protein sequences in roughly nine hours on a single GPU, making large-scale functional annotation of vast sequence collections realistically achievable.

Beyond large-scale classification, SPIN also enables more targeted analyses. If users wish to assess whether different occurrences of the same domain within a protein are associated with distinct functional classes, these occurrences can be extracted and provided to SPIN as independent sequences for classification. This enables the systematic investigation of whether repeated domains within a single protein contribute to different functional roles—a question that can be naturally explored using both ProfileView and SPIN.

Finally, SPIN assigns a confidence score to each input sequence for every functional class, reflecting the estimated probability of class membership, and proposes the class with the highest score. By avoiding the use of a fixed decision threshold, this score-based framework allows low-confidence or ambiguous predictions to be left unclassified, enabling users to select confidence cutoffs tailored to their specific application. This flexibility makes SPIN well-suited for large-scale exploratory analyses, such as domain-level annotation during initial functional screening, particularly in metagenomic contexts. However, accurate domain annotation remains inherently complex, as it often depends on the presence and interplay of multiple domains within a full-length protein. Dedicated annotation tools are specifically designed to account for such interactions, overlapping domains, and scoring hierarchies that prioritize the most reliable matches (Terrapon *et al.* 2009, Ochoa *et al.* 2011, Bernardes *et al.* 2016, Ugarte *et al.* 2018, Vicedomini *et al.* 2021). Therefore, when complete protein sequences are available, a comprehensive domain annotation using specialized tools is recommended following the initial screening performed with SPIN.

## Supplementary Material

vbag064_Supplementary_Data

## Data Availability

The data and the code underlying this article are available at: https://gitlab.lcqb.upmc.fr/andrea.mancini/SPIN.
