# Supplementary material for: Scaling the profile of life by function with SPIN
Source: Bioinform Adv. 2026 Feb 19;6(1):vbag064. doi: 10.1093/bioadv/vbag064 (PMC12970593; doi:10.1093/bioadv/vbag064)
Supplement: vbag064_Supplementary_Data [file vbag064_supplementary_data.pdf]

Supplementary Tables and Figures

Scaling the Profile of Life by Function with SPIN

Andrea Mancini, Vinh-Son Pho, Alessandro Bianchi, Gianluca Lombardi, Chujun Lyu, Alessandra Carbone

Model Classifier	Validation		Test	
	**Acc_w**	**$F1_{m-w}$**	**Acc_w**	**$F1_{m-w}$**
**Machine learning approaches**				
SVM	0.822	0.821	0.827	0.826
Gradient Boosting	0.704 ± 0.0	0.702 ± 0.0	0.690 ± 0.0	0.688 ± 0.0
Random Forest	0.71 ± 0.0	0.706	0.690 ± 0.0	0.686 ± 0.01
k-NN	0.693	0.685	0.705	0.699
**Deep Learning approaches**				
**One-hot encoding**				
1-D CNN	0.84 ± 0.0	0.84 ± 0.0	0.837 ± 0.1	0.837 ± 0.1
LSTM	0.654 ± 0.27	0.63 ± 0.30	0.639 ± 0.25	0.618 ± 0.28
**ESM2-35M - Average along hidden dimension**				
Baseline ESM2-35M Freezed	0.717 ± 0.01	0.715 ± 0.01	0.694 ± 0.01	0.691 ± 0.01
Baseline ESM2-35M Fine-tuned	0.893 ± 0.01	0.893 ± 0.01	0.887 ± 0.01	0.887 ± 0.01
ESM2-35M Freezed + 1-D CNN	0.878 ± 0.0	0.878 ± 0.0	0.876 ± 0.01	0.875 ± 0.01
ESM2-35M Fine-tuned + 1-D CNN	0.878 ± 0.01	0.879 ± 0.01	0.874 ± 0.02	0.874 ± 0.02
ESM2-35M Freezed + LSTM	0.888 ± 0.01	0.888 ± 0.01	0.887 ± 0.01	0.885 ± 0.01
ESM2-35M Fine-tuned + LSTM	0.891 ± 0.0	0.891 ± 0.0	0.887 ± 0.0	0.887 ± 0.0
ESM2-35M Freezed + Domain Span	0.782 ± 0.0	0.781 ± 0.0	0.789 ± 0.0	0.787 ± 0.0
**ESM2-35M Fine-tuned + Domain Span**	**0.913 ± 0.0**	**0.913 ± 0.0**	**0.906 ± 0.0**	**0.906 ± 0.0**

Table S1. Architecture performance comparison. The one-hot encoding representation, despite its simplicity and robustness, achieved strong performance (weighted accuracy and macro-weighted F1-score of 0.84). Inspired by **?**, this approach effectively handles variable sequence lengths. For ESM2-35M, fine-tuning of encoder layers did not necessarily improve performance, likely due to the smoothing operation that may have led to a loss of important learned features in the 1D-CNN model. In contrast, the baseline ESM2-35M benefited from fine-tuning, underscoring fine-tuning relevance in model optimization. The best overall results were obtained with the fine-tuned Domain Span architecture (**Figure 1**), although its performance remained comparable to that of the LSTM and CNN counterparts. Interestingly, the Domain Span module analysis shows that fine-tuning is necessary to achieve optimal performance. Results reported for the ESM2-35M model average three runs from different seeds.

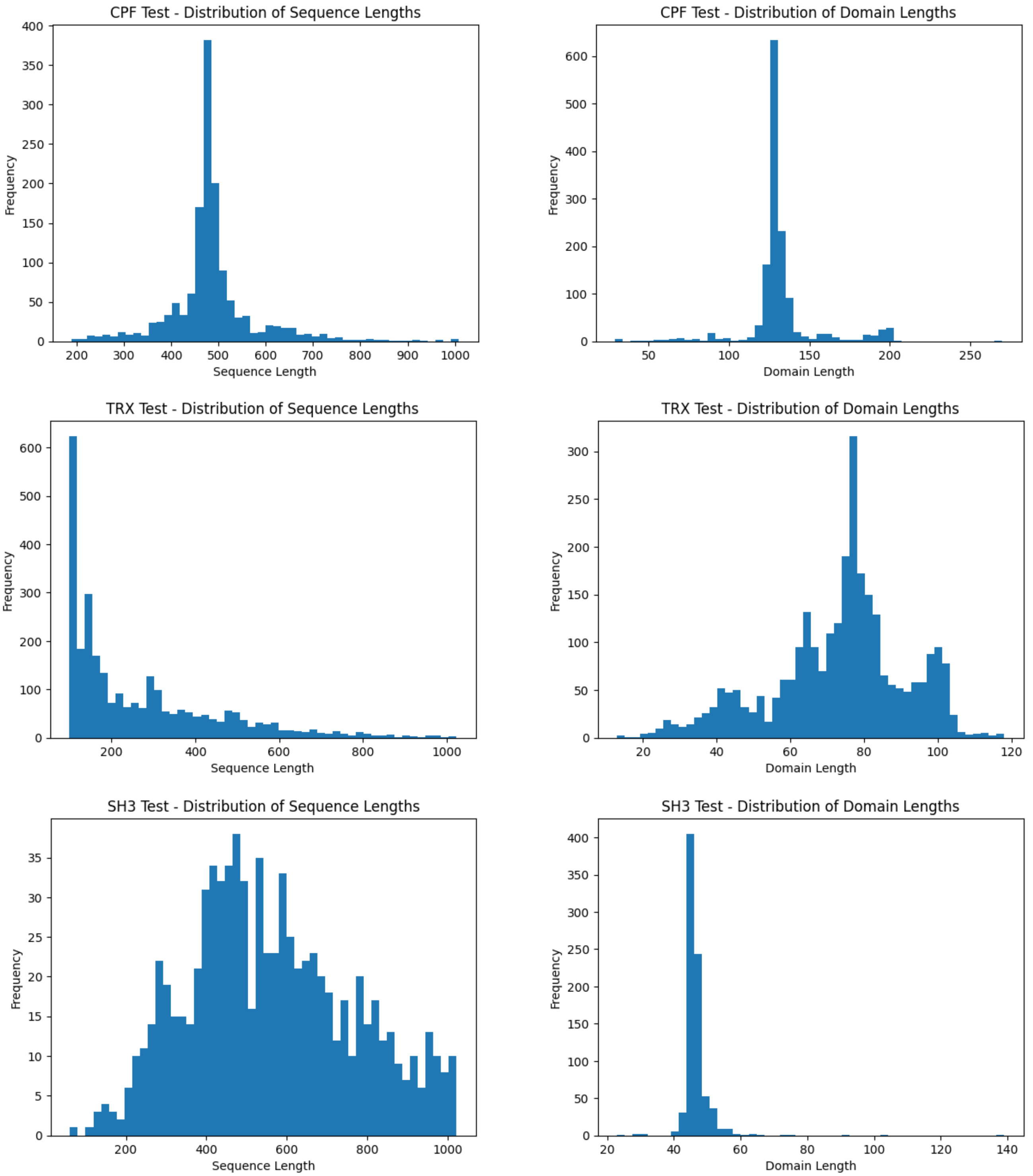

Fig. S1. Distribution of lengths of input sequences and their corresponding domains, for CPF, TRX and SH3 families, used during testing. Domain annotations (right) have been downloaded from UniProt.

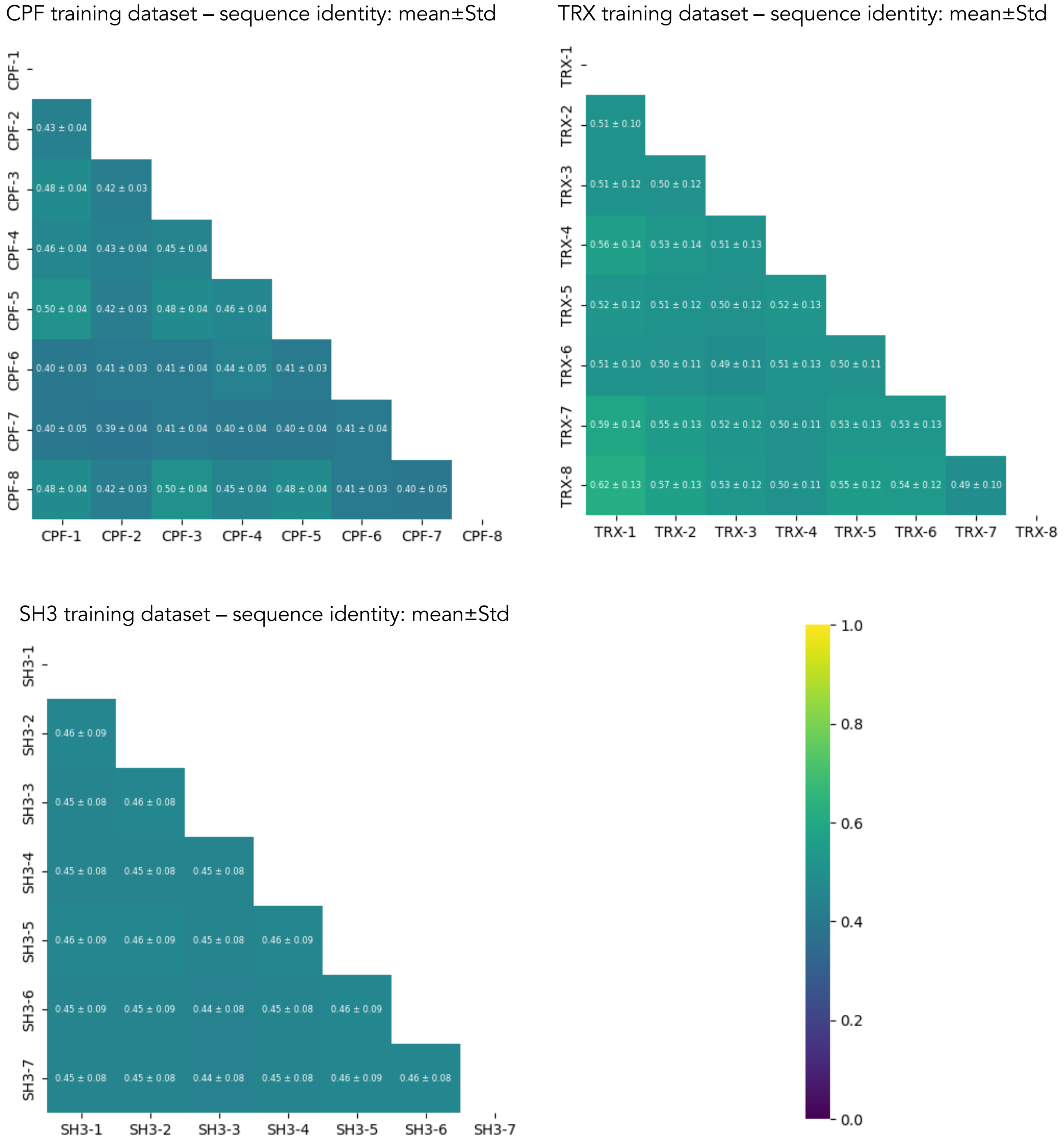

Fig. S2. Average sequence identity and standard deviation between training sequences in pairs of functional subclasses, for CPF, TRX and SH3 families. Values are calculated with Pairwise Aligner from BioPython library.

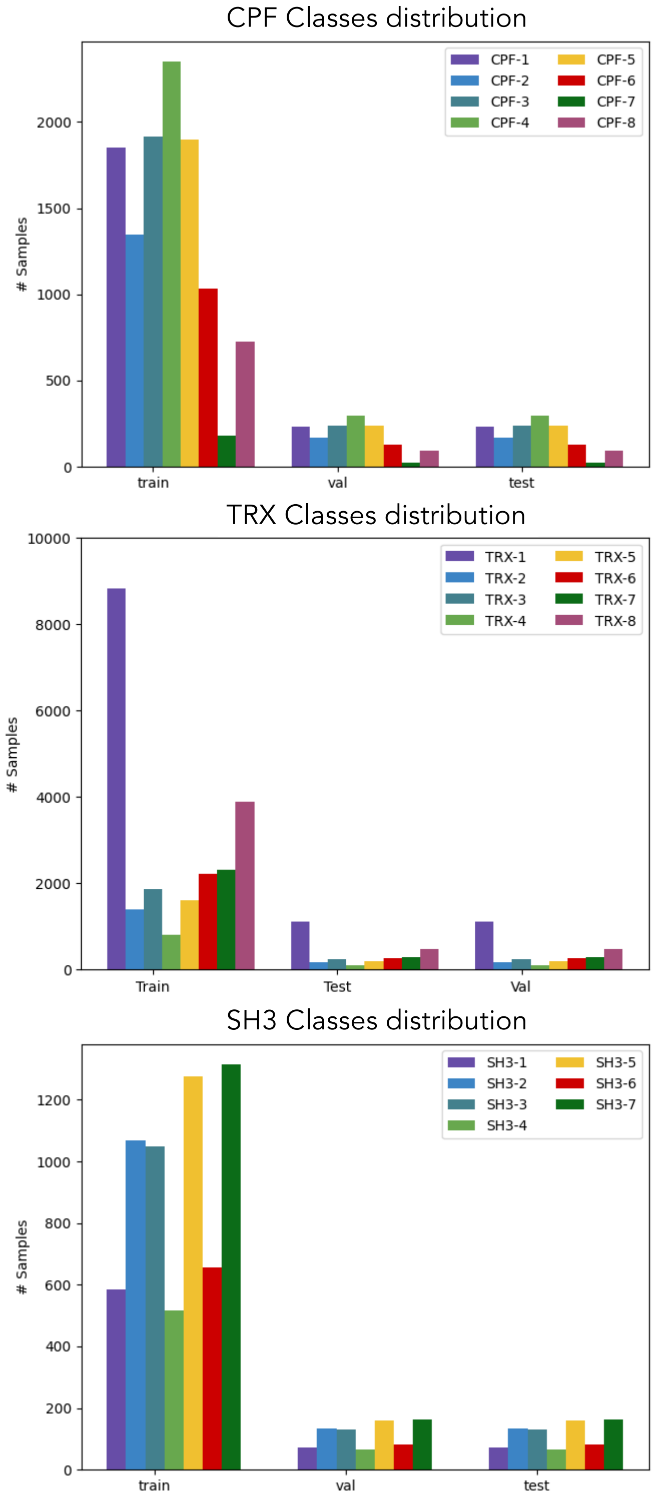

Fig. S3. Distribution of sequences in the training, validation, and testing datasets for CPF, TRX and SH3 families. Top: CPF-1, ..., CPF-8 label the eight functional CPF subclasses identified by ProfileView. **Middle:** TRX-1, ..., TRX-8 label the eight functional TRX subclasses identified by ProfileView. **Bottom:** SH3-1, ..., SH3-7 label the seven functional SH3 subclasses identified by ProfileView.

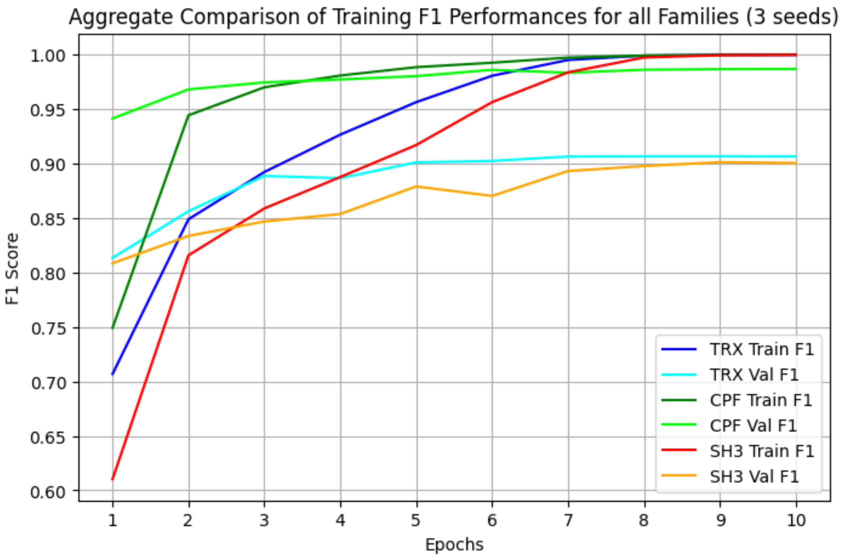

Fig. S4. SPIN performance comparison on TRX, SH3 and CPF families. $F1_{\text{m-w}}$ scores are computed on the training and validation sets across the TRX (blue tones), SH3 (red tones), and CPF (green tones) protein families. Values at each epoch represent averages over three random seeds.

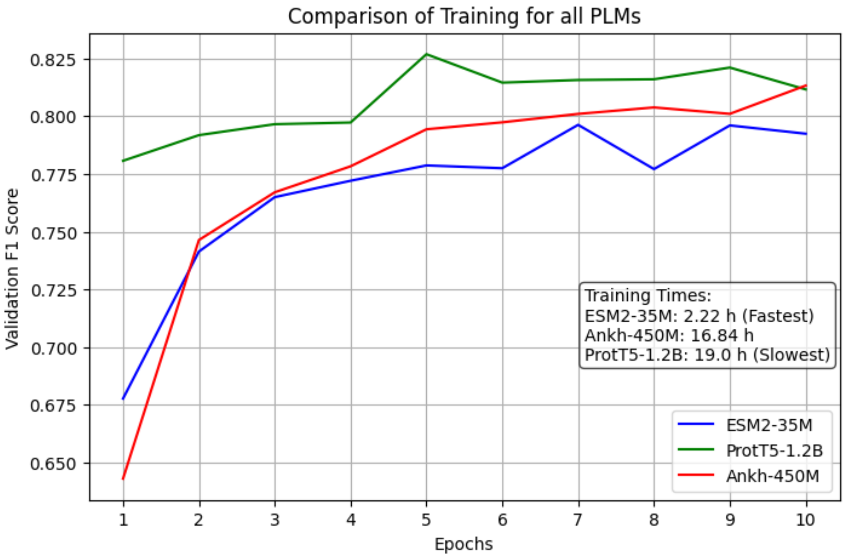

Fig. S5. Performance comparison of 3 different PLMs for the SPIN backbone: ESM2-35M, ProtT5 and Ankh-base. $F1_{\text{m-w}}$ score is computed on the validation set across 10 epoches (y-axis). ESM2-35M achieves very competitive results while being 8x faster.

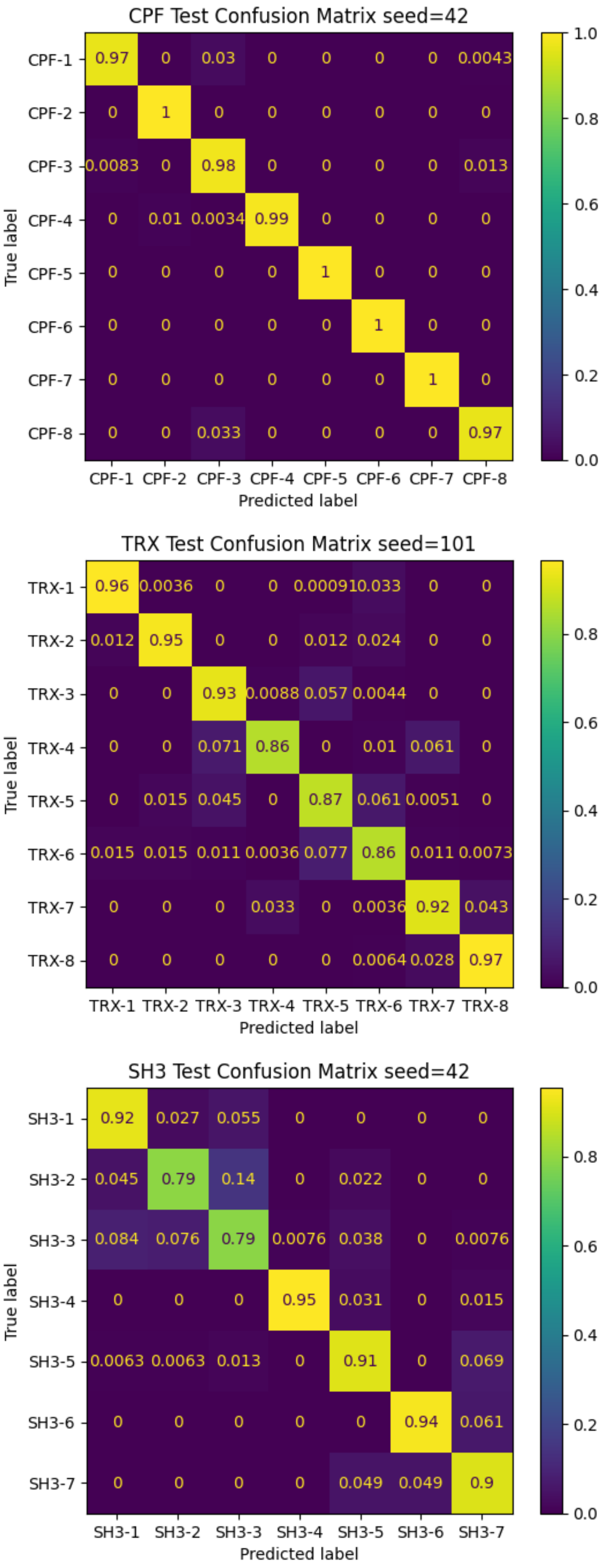

Fig. S6. Recall and confusion matrices for CPF, TRX and SH3. Recall values are reported on the diagonal for each subclass. Each row is normalized by the total number of true sequences in the class, representing the fraction of correctly predicted sequences. **Top:** CPF **Middle:** TRX **Bottom:** SH3.

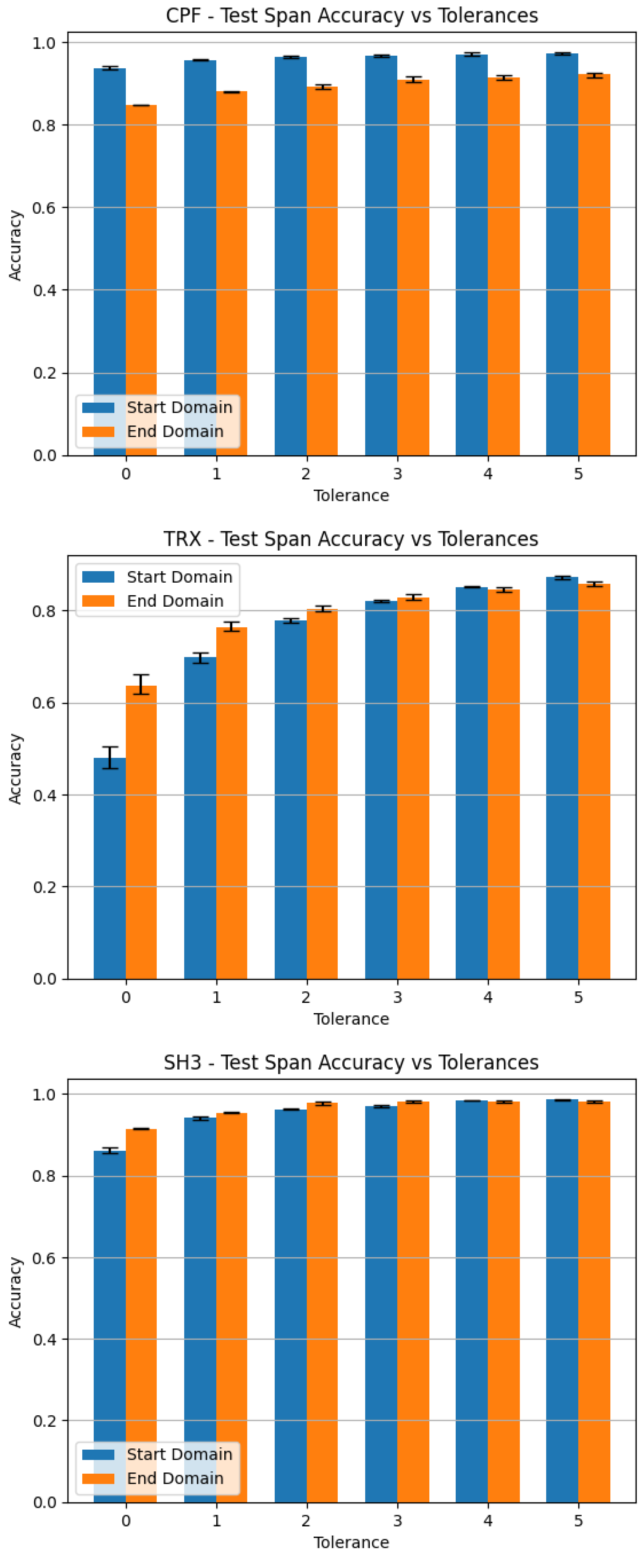

Fig. S7. Accuracy for the domain span test for the CPF, TRX and SH3 families. Accuracy was computed across a range of tolerance values, averaged over three random seeds. Error bars indicate the deviation across seeds. **Top:** CPF **Middle:** TRX **Bottom:** SH3.

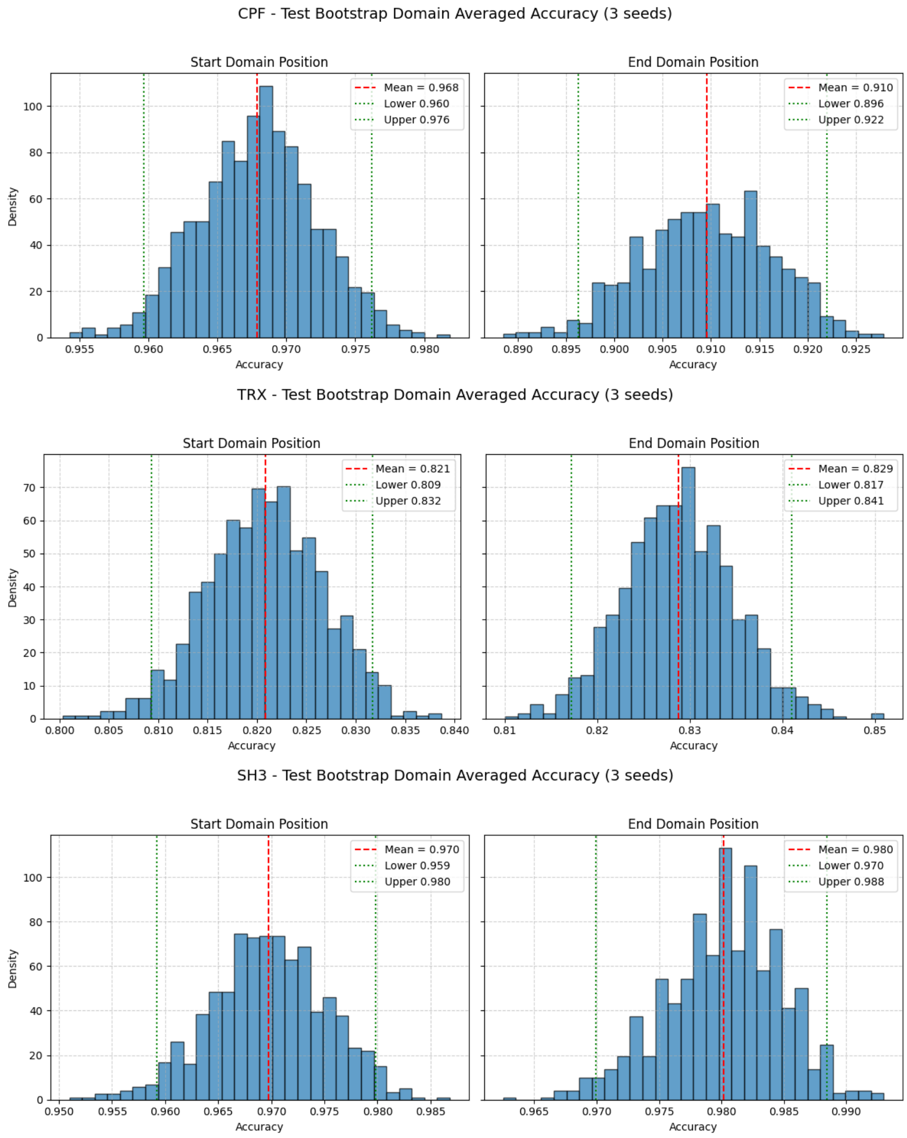

Fig. S8. Bootstrap evaluation of the span domain predictions. Bootstrap analysis was performed by repeatedly resampling the test set with replacement for 1000 times, and computing the domain boundary accuracy on test set within a tolerance of ±3 residues, averaged over three random seeds to ensure robustness. A Confidence Interval (CI) of 95% around mean value was considered. **Top:** CPF sequences; **Middle:** TRX sequences; **Bottom:** SH3 sequences

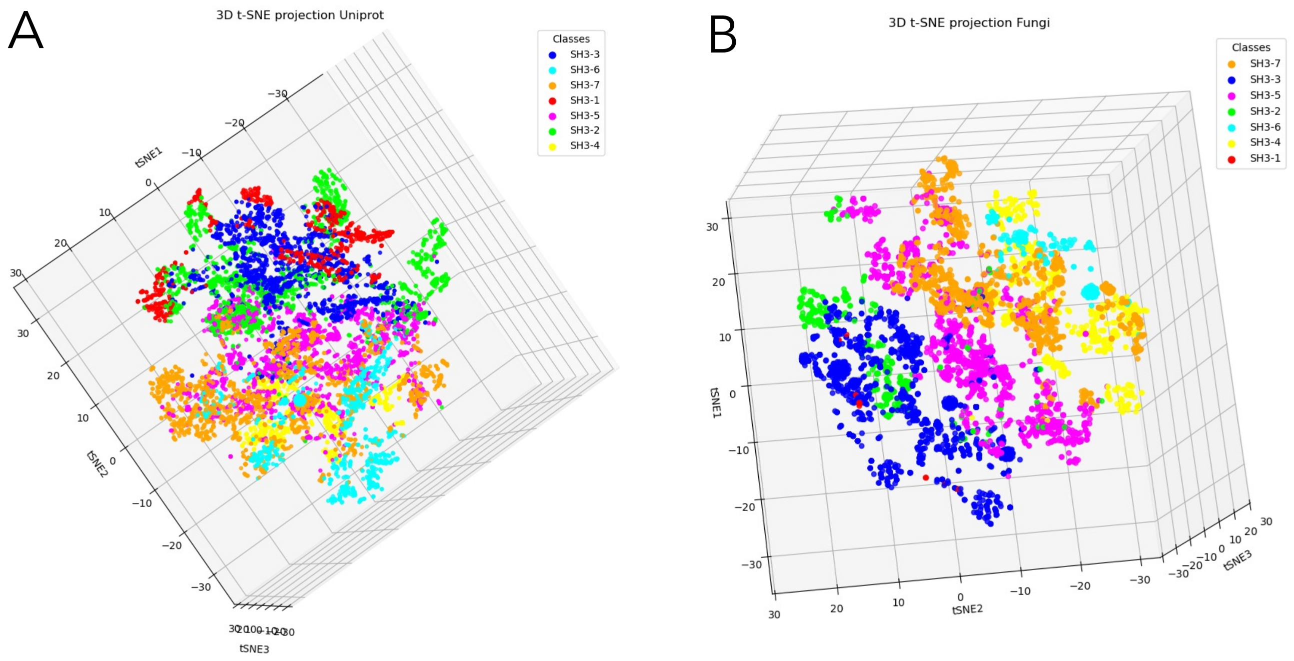

Fig. S9. Classification space of the SH3 domains in the UniProt and Fungi datasets. t-SNE projections of the multidimensional functional spaces constructed with ProfileView: (A) UniProt dataset and (B) Fungi dataset. Sequences are colored after SPIN classification. Color legend as in **Figure 4A**.

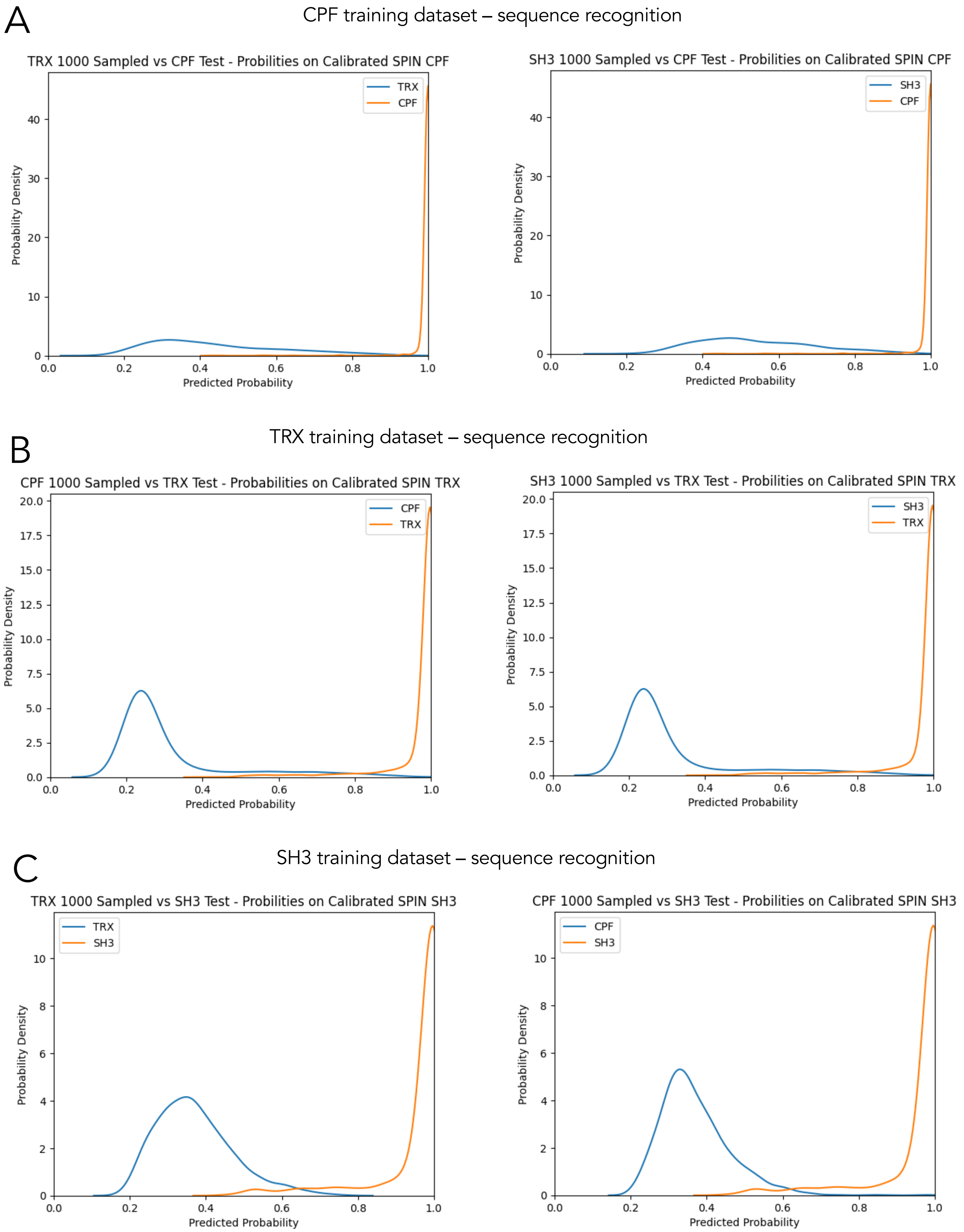

Fig. S10. Score distributions in SPIN classification for homologous and non homologous sequences. A. Classification of TRX (left, blue) and SH3 (right, blue) sequences by SPIN trained on the CPF protein family. Score distribution for CPF test sequences in orange. B. Classification of CPF (left, blue) and SH3 (right, blue) sequences by SPIN trained on the TRX protein family. Score distribution for TRX test sequences in orange. C. Classification of TRX (left, blue) and CPF (right, blue) sequences by SPIN trained on the SH3 protein family. Score distribution for SH3 test sequences in orange.

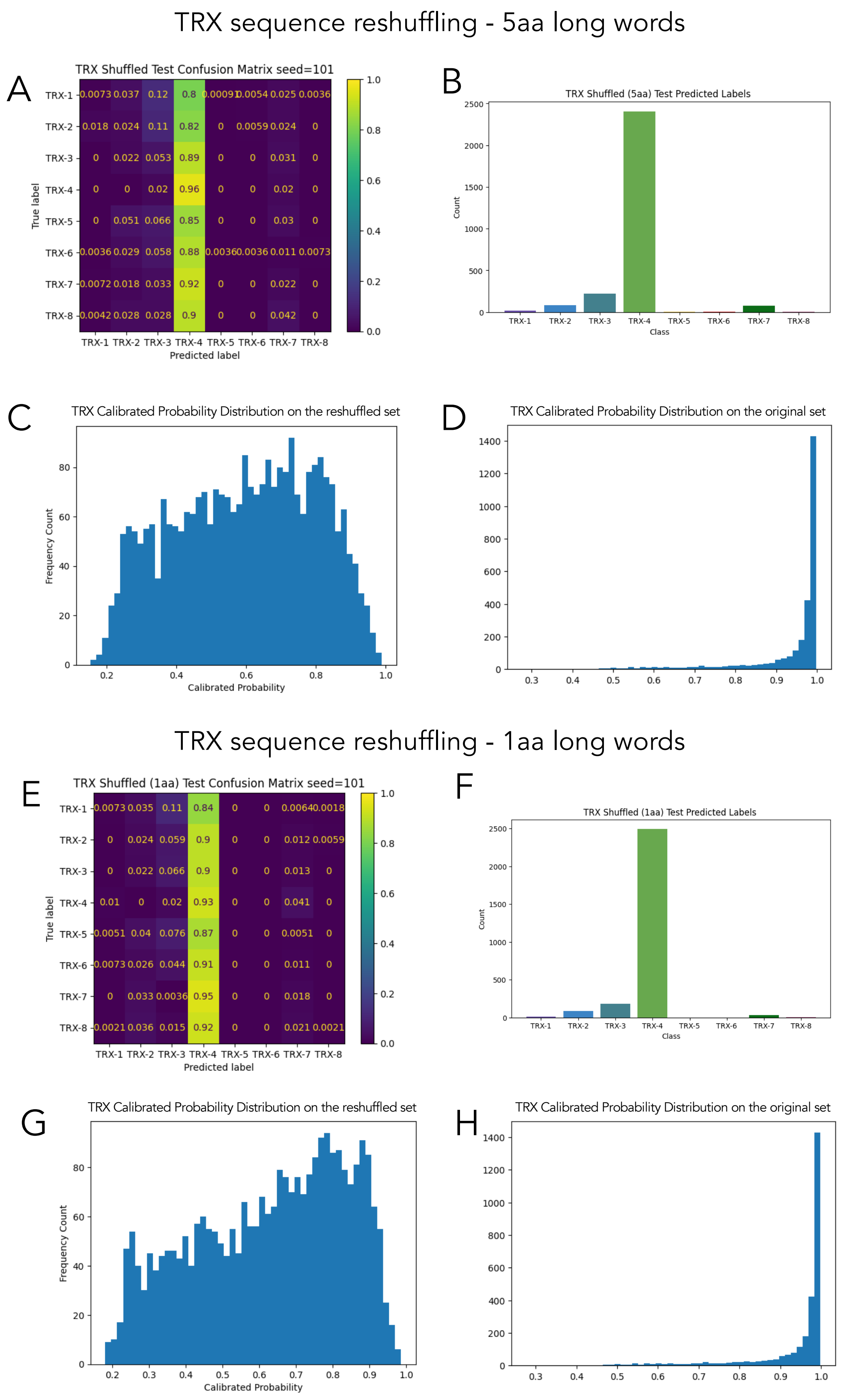

Fig. S11. Evaluation of SPIN , trained on the TRX family, on random sequences. SPIN was evaluated on randomized TRX test sequences generated by reshuffling contiguous segments of length 5 (top) and 1 (bottom) amino acids. A,E. Confusion matrix across the eight TRX classes. B,F. Distribution of shuffled TRX sequences classified in the eight TRX classes. C,G. Distribution of calibrated probability scores for the shuffled TRX sequences. D,H. Distribution of calibrated probability scores for the original set of TRX sequences. Compare to C,G.
